# Endophytic colonization of *Beauveria peruviensis* and its antagonistic activity against *Neopestalotiopsis mesopotamica* in blueberry

**DOI:** 10.3389/fpls.2026.1829417

**Published:** 2026-05-19

**Authors:** Isabel Barrera-Merino, Marisol Vargas, Macarena Gerding González, Jean Franco Castro, Viviana Cisterna-Oyarce, Ernesto A. Moya-Elizondo, Lorena Barra-Bucarei

**Affiliations:** 1 Facultad de Ingeniería y Negocios, Universidad Adventista de Chile, Chillán, Chile; 2 Departamento de Producción Vegetal, Facultad de Agronomía, Universidad de Concepción, Chillán, Chile; 3 Laboratorio de Bioprocesos, Instituto de Investigaciones Agropecuarias, INIA Quilamapu, Chillán, Chile

**Keywords:** biocontrol, highbush blueberry, Pestalotioid species, phytopathogens, *Vaccinium corymbosum* L.

## Abstract

Chile is one of the leading blueberry producers in the Southern Hemisphere. The environmental conditions in the central-southern regions of the country favor infection by *Neopestalotiopsis* species, which cause blight, cankers, and dieback that cause the death or decline of plant tissues, especially stems. The use of biocontrol agents represents a promising strategy for managing these fungal diseases. This study evaluated the endophytic colonization capacity, persistence, and antagonistic activity of *Beauveria peruviensis* against *Neopestalotiopsis mesopotamica* in ‘Duke’ blueberry plants. Six strains of *B. peruviensis* were sprayed on blueberry plants (1×10^6^ conidia mL^-1^) to assess colonization and persistence in leaves, stems and roots at 4, 8, and 12 weeks post-inoculation. The antagonistic activity of *B. peruviensis* strains against *N. mesopotamica* was evaluated both *in vitro*, using dual culture assays, and *in vivo*, through co-inoculation in blueberry stems. All six strains demonstrated endophytic colonization in leaves (16% to 64%) and stems (20% to 32%); however, none were recovered from the roots. The persistence of *B. peruviensis* strains varied significantly among treatments. While all strains exhibited endophytic colonization at four weeks post-inoculation, only strains RGM 547, RGM 557, and RGM 570 persisted through 12 weeks. *In vitro*, all six strains exhibited inhibition of radial growth, ranging from 46.9% to 54.7%. *In vivo*, strains RGM 547, RGM 557, and RGM 570 significantly reduced stem necrosis length compared to the positive control inoculated with *N. mesopotamica* alone. A significant inverse correlation was observed between endophytic colonization and stem necrosis severity. The results suggest that *B. peruviensis* is a sustainable alternative to synthetic fungicides for managing stem blight disease in blueberry cultivation.

## 1 Introduction

Blueberry (*Vaccinium corymbosum* L.) is cultivated worldwide for its fruits, which are rich in of antioxidants such as ascorbic acid and phenolic acids, known to help mitigate the development of various human diseases (Ji et al., 2019). Chile is the second largest exporter of blueberries in the Southern Hemisphere and, together with Peru, serves as a key off-season supplier to Northern Hemisphere markets (Macha Huamán et al., 2023; Trejo-Pech et al., 2024). The total area planted with blueberries in Chile is approximately 17,000 hectares (ha), with the highest concentrations found in the central-southern regions, including Maule (5,625 ha) and Ñuble (4,143 ha), as well as the southern regions of Araucanía (1,899 ha) and Los Ríos (1,802 ha) (ODEPA, 2024). In these regions, spring rainfall increases environmental humidity, which favors the development of fungal and bacterial phytopathogens (Moya-Elizondo, 2020; Lahlali et al., 2024). These pathogens can adversely affect plant development, reduce productive lifespan, and compromise both fruit quality and quantity (Espinoza et al., 2008).

Blueberry plants are affected by various pathogens from the genera *Neofusicoccum*, *Diaporthe* and *Neopestalotiopsis*, which may occur individually or concurrently within the same plant, causing significant damage (Espinoza et al., 2009, 2008; Elfar et al., 2013). Species of the genus *Neopestalotiopsis*, a subgenus of *Pestalotiopsis sensu lato* within the Sporocadaceae family, are characterized by shared asexual morphological features and are distributed across temperate and tropical regions (Liu et al., 2022; Maharachchikumbura et al., 2014). In blueberry plants, pestalotioid are among the main causal agents of blight, cankers, and dieback of stems (Barrera-Merino et al., 2025; Dietsch et al., 2025; Borrero et al., 2018; Lee et al., 2019; Rodríguez-Gálvez et al., 2020; Jevremović et al., 2022; Santos et al., 2022; Shi et al., 2017; González et al., 2012; Espinoza et al., 2008; Chen et al., 2016). Numerous studies have documented the presence of *Neopestalotiopsis* species associated with these symptoms across several countries, including Chile (Espinoza et al., 2008; Barrera-Merino et al., 2025), the United States (Dietsch et al., 2024), Uruguay (González et al., 2012), Peru (Rodríguez-Gálvez et al., 2020), China (Shi et al., 2017; Chen et al., 2016), Korea (Lee et al., 2019), Spain (Borrero et al., 2018), Portugal (Santos et al., 2022), and Serbia (Jevremović et al., 2022). Reported pestalotioid species affecting blueberry include *N. clavispora* (Borrero et al., 2018; González et al., 2012; Espinoza et al., 2008; Chen et al., 2016; Lee et al., 2019; Jevremović et al., 2022), *Pestalotiopsis neglecta* (Espinoza et al., 2008), *N. rosae* (Rodríguez-Gálvez et al., 2020; Dietsch et al., 2024), *N. scalabiensis*, *N. vacciniicola*, *N. vaccinii*, *P. chamaeropis*, and *P. biciliata* (Santos et al., 2022). Recently, *N. mesopotamica* was reported for the first time infecting *V. corymbosum* in Chile (Barrera-Merino et al., 2025).

Stem blight is considered one of the most destructive diseases affecting blueberry production worldwide (Rodríguez-Gálvez et al., 2020; Jevremović et al., 2022). It causes leaf chlorosis, complete branch dieback in new shoots, dark brown stem girdling and abundant black conidia on infected stems (Rodríguez-Gálvez et al., 2020; Santos et al., 2022; Barrera-Merino et al., 2025). Effective management of this disease requires integrated control strategies, including the removal of plant debris, improvement of air circulation, sanitary pruning, prevention of insect damage, and application of chemical fungicides (Zhang et al., 2024; Ru et al., 2022; Maharachchikumbura et al., 2014). However, in recent years, there has been increasing interest in the use of biological control agents as an alternative to synthetic chemical fungicides, which face limitations such as the development of pathogen resistance, reduction of beneficial microorganisms, soil and water contamination, and risks to human health (Bailey et al., 2010; Attia et al., 2022; Rondot and Reineke, 2019).

Entomopathogenic fungi have been extensively studied for their role in insect parasitism (Sharma et al., 2023; Lozano-Tovar et al., 2013, 2017; Bamisile et al., 2019; Barra-Bucarei et al., 2020). However, they have also demonstrated additional functions as plant endophytes (Barra-Bucarei et al., 2019; Bamisile et al., 2019, 2020; Quesada-Moraga, 2020; Jaber and Ownley, 2018; Vega, 2008). Some of these fungi can act as antagonists against plant pathogens or colonize plants to promote growth (Ownley et al., 2008; Vega et al., 2009; Jaber and Ownley, 2018; Barra-Bucarei et al., 2019; Bamisile et al., 2020).The genus *Beauveria* (Ascomycota: Hypocreales) is one of the most extensively studied entomopathogenic fungi for its endophytic capabilities (Parsa et al., 2013; Jaber, 2015). Globally, this fungus naturally occurs in soils and can artificially colonize various plant tissues such as leaves, stems, flowers, and roots, when applied through foliar sprays, seed soaking, or soil application (Arnoldi et al., 2022; Krell et al., 2017; Quesada-Moraga, 2020). Successful colonization has been demonstrated in a wide range of plant species, including grasses (Sánchez-Rodríguez et al., 2018; Altaf et al., 2023); solanaceous crops (Ownley et al., 2004; Barra-Bucarei et al., 2019; Silva et al., 2020; Wei et al., 2020; Toffa et al., 2021); opium poppy (Quesada-Moraga et al., 2006); legumes (Jaber and Enkerli, 2016; Ren et al., 2024); and tropical ornamental species (Lopez and Sword, 2015; Gómez-Vidal et al., 2006; Akello et al., 2007; Posada and Vega, 2006; Posada et al., 2007; Posada and Vega, 2005). Moreover, the antagonistic effects of *Beauveria* species have been studied against various pathogens, such as *Fusarium oxysporum* and *Macrophomina phaseolina* in soybean (Russo et al., 2024), *Rhizoctonia solani* in rice (Deb et al., 2023) and tomato (Ownley et al., 2004), *Pythium myriotylum* in tomato (Deb and Dutta, 2021) and cotton (Ownley et al., 2008), *Plasmopara viticola* in grapevine (Jaber, 2015), *F. oxysporum* in chili pepper and *F. culmorum* in wheat (Jaber and Ownley, 2018), and *Botrytis cinerea* in tomato and chili pepper (Barra-Bucarei et al., 2019). The antagonistic activity of *Beauveria* spp. can occur through direct mechanisms such as mycoparasitism, competition, or antibiosis via the production of secondary metabolites (e.g., beauvericin, beauverolides, bassianolides, bassianolone, bassianin, bassiacridin, oosporein, cyclosporin, and oxalic acid) that exhibit antibacterial, antifungal, cytotoxic, and insecticidal activities (Russo et al., 2024; Wang et al., 2021). Alternatively, indirect antagonism can occur through the induction of plant systemic resistance, stimulation of plant secondary metabolites, and promotion of plant growth (Jaber and Ownley, 2018; Qin et al., 2021).

In Chile, more than 600 strains of *Beauveria* spp. have been collected and are available in the Chilean Collection of Microbial Genetic Resources (CChRGM) of the Microbial Genetic Resources Bank (BRGM) at the Institute of Agricultural Research (INIA, Quilamapu). Some of these strains have demonstrated high pathogenicity against insect pests and phytopathogens of national importance (France et al., 2000; Barra-Bucarei et al., 2019, 2020). However, the use of native strains of *Beauveria peruviensis* as a biological control agent against *Neopestalotiopsis mesopotamica*, recently described in Chile as a pathogen in blueberry plants (Barrera-Merino et al., 2025), has not yet been investigated. This strategy could offer a more sustainable alternative to chemical fungicides by helping reduce plant losses in nurseries and promoting the establishment of healthy plants in cultivated fields. Based on this background, the objective of this research was to determine the colonization capacity and endophytic persistence of *B. peruviensis* in blueberry plants, and to evaluate its antagonistic activity against *N. mesopotamica* in this berry host.

## 2 Materials and methods

### 2.1 Biological and microbiological material

Blueberry nursery plants of the Duke cultivar, previously cultivated *in vitro*, were obtained from the Proplant Plant Propagation Center of the Faculty of Agronomy, Universidad de Concepción. Cryopreserved native fungal strains of *B. peruviensis* RGM 547, RGM 557, RGM 570, RGM 644, RGM 657, and RGM 731 (**Table 1**), previously reported as endophytes in tomato and chili pepper (Barra-Bucarei et al., 2019), along with the cryopreserved pathogenic strain *N. mesopotamica* RGM 3491 (Barrera-Merino et al., 2025), were obtained from the Chilean Collection of Microbial Genetic Resources (CChRGM) at the Microbial Genetic Resources Bank (BRGM) of INIA Quilamapu, located in Chillán, Ñuble Region, Chile.

**Table 1:** List of *Beauveria peruviensis* isolates, including strain code assigned in Microbial bank collection, collection site and origin, used to assess colonization capacity, endophytic persistence in blueberry, and antagonistic activity against *Neopestalotiopsis mesopotamica*.

Strain Code	Collection Location	Origin
**RGM 547**	Santa Barbara, Biobío Region, Chile	Natural meadow soil
**RGM 557**	Los Lagos, Los Lagos Region, Chile	Natural meadow soil
**RGM 570**	Molina, Maule Region, Chile	Natural meadow soil
**RGM 644**	Icalma, Araucanía Region, Chile	Natural meadow soil
**RGM 657**	Puerto Ibañez, Aysen Region of General Carlos Ibáñez del Campo, Chile	Arable land, cultivation of *Vitis vinifera* fruits
**RGM 731**	Cisnes River, Aysen Region of General Carlos Ibáñez del Campo, Chile	Natural meadow soil

### 2.2 Endophytic colonization of *Beauveria peruviensis* strains in blueberry plants

#### 2.2.1 Preparation of *Beauveria peruviensis* inoculum

To obtain inoculum, cryopreserved conidia from the *B. peruviensis* strains described in **Table 1**, stored in the CChRGM, were inoculated following the protocol described by Barra-Bucarei et al. (2019). The strains were extracted using a sterile inoculation loop in a sufficient quantity to ensure uniform seeding on Petri plates containing potato dextrose agar (PDA, Difco™) with 150 mg L^-1^ of chloramphenicol. The plates were then incubated in the dark at 25 ± 2 °C for 10 days. After the incubation period, conidial suspensions were prepared in test tubes with 10 mL of sterile distilled water and 0.01% (v/v) Tween 80. The suspensions were diluted to achieve a final concentration of 1 × 10^6^ conidia mL^-1^, determined using a Neubauer counting chamber (BOECO, Germany).

#### 2.2.2 Inoculation of blueberry plants with *Beauveria peruviensis* strains

Five-month-old blueberry plants (cv. Duke) were maintained in 1.25 L pots with a substrate composed of a sterilized mixture of perlite, peat, compost, and vermiculite (2:2:2:1). The substrate was sterilized twice in an autoclave at 120 °C and 115 psi for 1 hour.

Plant inoculation was performed on the foliage using a handheld atomizer, applying 25 mL of a suspension containing 1 × 10^6^ conidia mL^-1^ of each fungal strain. For the untreated control, sterile distilled water with 0.01% Tween 80 was used. To prevent conidia from running off into the substrate, aluminium foil was placed over the pots. To maintain humidity (≈ 90%) and promote stomatal opening, inoculated plants were covered with cellophane bags for 24 hours (Bamisile et al., 2020; Posada et al., 2007). The plants were then incubated for 30 days in growth chambers at 25 ± 2 °C with a photoperiod of 16 hours of light and 8 hours of darkness. Plants were watered uniformly with distilled water every two days.

A completely randomized design was used, with each plant in a single pot as an experimental unit. Five replicates were considered for each *B. peruviensis* strain, along with a control treatment without fungal inoculation, totalling 35 plants in the experiment.

#### 2.2.3 Re-isolation of endophytic *Beauveria peruviensis* strains from inoculated blueberry tissues.

After 30 days post-inoculation, plants were removed from the pots, and the roots were washed with tap water. Subsequently, the plants were placed inside a laminar flow cabinet, where the roots, stems, and leaves were carefully separated. Each tissue sample was disinfected by immersion in 70% ethanol for 15 seconds, followed by 1.5% NaOCl for 3 minutes, and finally rinsed again in 70% ethanol for 15 seconds. The samples were then rinsed three times with sterile distilled water for 1 min each and left to dry on sterile absorbent paper (Barra-Bucarei et al., 2019).

Once disinfected, 10 sub-samples were taken from each plant tissue (root, stem, and leaves), totaling 30 tissue segments per plant. Root and stem segments were cut to 10 mm in length, while leaf segments measured 6 mm². The segments were placed on Petri dishes containing Noble agar medium with 150 mg L^-1^ of chloramphenicol and incubated in the dark at 25 ± 2°C for 30 days (Barra-Bucarei et al., 2019).

After incubation, the segments were examined for fungal growth emerging from within the tissues, and the percentage of endophytic colonization (PEC) was recorded. To confirm the presence of *Beauveria*, morphological identification was performed by transferring small agar samples (1 cm²) with mycelial growth onto microscope slides. The fungal structures were observed under an optical microscope (Leica Microsystems, DM500, Germany) at 40× magnification and identified using taxonomic keys (Watanabe, 2002).

Re-isolation was conducted from each plant (experimental unit), with five replicates taken for each plant tissue segment (leaf, stem, and root) across all seven treatments.

### 2.3 Evaluation of the persistence of *Beauveria peruviensis* strains in blueberry plants

One-year-old ‘Duke’ blueberry plants were used and maintained under the same conditions as previously described. Inoculation was carried out by applying 50 mL of a conidial suspension (1 × 10^6^ conidia mL^-1^) to the foliage of each plant. The same precautions described above were taken; aluminium foil was placed over the pots to prevent conidia runoff into the substrate, and cellophane bags were used to cover the plants for 24 hours to promote fungal colonization (Bamisile et al., 2020; Posada et al., 2007). After inoculation, the plants were maintained at 25 ± 2°C for 30 days under a 16-hour light/8-hour dark photoperiod. Watering was carried out every two days using a uniform amount of distilled water. The experiment followed a completely randomized design with five plant replicates for each of the six *Beauveria* strains, as well as a control treatment that received only sterile distilled water with 0.01% Tween 80. Each plant, housed in an individual pot, was considered an experimental unit, totalling 35 plants in the trial.

The persistence of the fungal strains (**Table 1**) was assessed at 30, 60, and 90 days post-inoculation (corresponding to four, eight, and twelve weeks). PEC was determined using the previously described re-isolation methodology, focusing exclusively on leaf tissue fragments (Bamisile et al., 2019).

### 2.4 *In vitro* antagonism of endophytic Beauveria peruviensis strains against Neopestalotiopsis mesopotamica

The antagonistic activity of the six *B. peruviensis* strains (**Table 1**) against *N. mesopotamica* was evaluated using the dual culture method. For this assay, 5 mm diameter mycelial discs of *Beauveria* were placed on 90 mm Petri dishes containing 20 mL of PDA supplemented with 150 mg L^-1^ of chloramphenicol, positioning the discs 1.5 cm from the edge of the plate. After five days of incubation, a 5 mm diameter mycelial disc of *N. mesopotamica* (RGM 3491) was placed at 55 mm from the *Beauveria* disc. Control plates containing only the pathogen were prepared in parallel.

The plates were incubated in the dark at 25 ± 2°C until the pathogen reached the edge of the Petri dish in the control plates. Colony radii (mm) were measured for each treatment using a digital caliper (Platinum, China) to determine the percentage of radial growth inhibition of pathogen (PRGIP) using the following formula: PRGIP = [(R1−R2)/R1] × 100, where R1 is the colony radius of *N. mesopotamica* growing alone in the control, and R2 is the colony radius of *N. mesopotamica* in competition with the endophytic *Beauveria* strain (Ezziyyani et al., 2007). Each Petri dish was considered an experimental unit, with five replicates per treatment. The entire experiment was conducted twice to confirm the results.

### 2.5 *In vivo* antagonistic effect of *Beauveria peruviensis* strains against *Neopestalotiopsis mesopotamica* in blueberry plants

#### 2.5.1 Inoculum preparation

The inoculum of the six *B. peruviensis* strains (**Table 1**) was prepared at a concentration of 1 × 10^6^ conidia mL^-1^ in tubes containing sterile distilled water and 0.01% Tween 80 (Difco™) (Barra-Bucarei et al., 2019). The *N. mesopotamica* inoculum was cultured for seven days in PDA medium (Santos et al., 2022; Espinoza et al., 2008) before use.

#### 2.5.2 Inoculum application

Two experiments were conducted in parallel: one using five-month-old ‘Duke’ blueberry plants and another using one-year-old plants of the same cultivar. The blueberry plants were obtained through *in vitro* propagation. The plants were established in individual 1.25 L pots filled with a substrate mixture of perlite, peat, compost, and vermiculite (2:2:2:1), which was sterilized twice in an autoclave at 120°C and 115 psi for 1 hour.

Five-month-old plants were inoculated with 25 mL of the conidial suspension of each *Beauveria* strain, while one-year-old plants received 50 mL of the suspension. The plants were subsequently covered with a cellophane bag for 24 hours, as previously described. Control plants were treated with sterile distilled water using the same volumes as the conidial suspensions.

*Neopestalotiopsis mesopotamica* was inoculated five days after the application of the antagonistic fungi, following the methodology described by Barrera-Merino et al. (2025). Briefly, healthy stems were selected, and a sterile cut of approximately 0.5 cm² was made to expose the cambium. A 5 mm diameter disc of pathogen mycelium was then inserted. For the control treatment, PDA discs without the pathogen were used. The inoculation site was sealed with Parafilm^®^ to prevent rapid dehydration and contamination of the wounded tissue.

The six *Beauveria* strains, along with two control treatments (a positive control, C+, and a negative control, C−) treated with sterile distilled water, were considered as treatments. The negative control (C−) was not inoculated, while the positive control (C+) was inoculated only with the *N. mesopotamica* isolate RGM 3491. The eight treatments were arranged in a completely randomized design with five replicates per treatment. Plants were maintained at an approximate temperature of 25 ± 2°C for 30 days, under a 16/8-hour photoperiod (day/night) and regular irrigation to prevent drought stress.

Stem severity was assessed 30 days after inoculation with the *B. peruviensis* strains. Necrosis length (mm) was measured from the inoculation point of *N. mesopotamica* using a digital caliper (Espinoza et al., 2008; Millas et al., 2023; Rodríguez-Gálvez et al., 2020). In parallel, re-isolation of *Beauveria* strains from leaves and stems was performed, as previously described, to determine the correlation between the presence of the antagonist and the damage observed following *N. mesopotamica* inoculation.

### 2.6 Statistical analysis

All experiments were conducted using a completely randomized design. Data normality was assessed through residual analysis using the Shapiro-Wilk test, and homogeneity of variance was evaluated using absolute residuals (abs. residues) within the framework of analysis of variance (ANOVA). When the data met parametric assumptions, an ANOVA was performed, followed by means comparisons using Tukey’s test at a significance level of *P* < 0.05. For variables that did not meet parametric assumptions, nonparametric tests such as the Kruskal-Wallis test followed by multiple pairwise comparisons, and the Friedman test, were applied (*P* < 0.05). Additionally, correlation analyses were conducted using the nonparametric Spearman test (*P* < 0.05). All statistical analyses were performed using InfoStat software (Di Rienzo et al., 2008).

## 3 Results

### 3.1 Endophytic colonization of *Beauveria peruviensis* strains in blueberry plants

**Figure 1:**
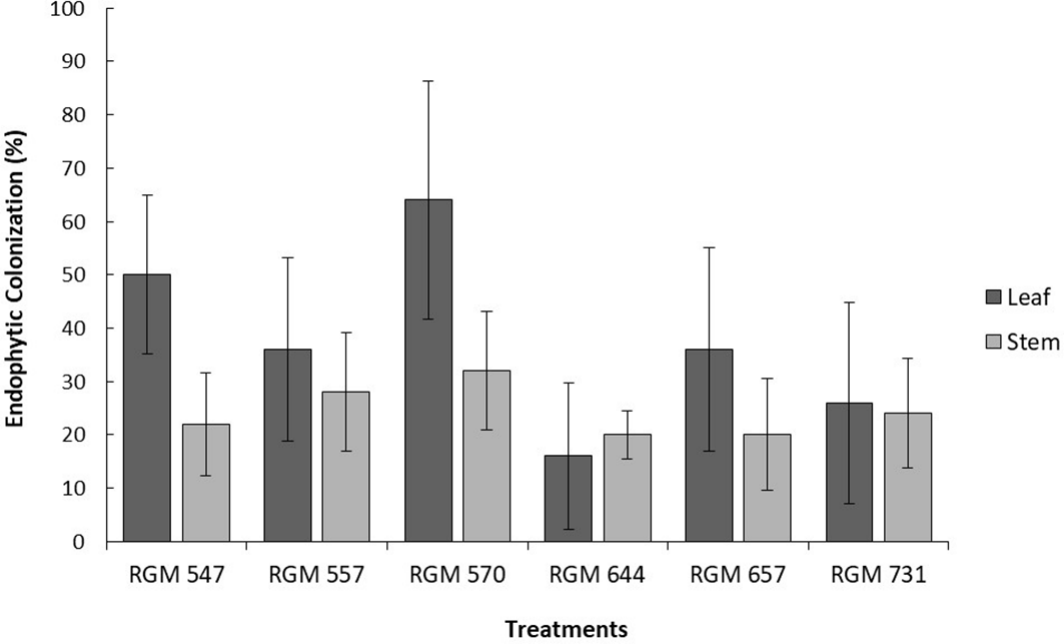
Percentages of endophytic colonization by different *Beauveria peruviensis* strains observed in the leaves and stems of five-month-old 'Duke' blueberry plants, 30 days after foliar inoculation. Columns represent treatment means, and the bars above them indicate the standard error for each treatment

All six evaluated *B. peruviensis* strains successfully colonized internally different tissues of blueberry plants (**Figure 1**). The highest percentages of endophytic colonization were observed in the leaves, ranging from 16% to 64%, while colonization in the stems ranged from 20% and 32%. No statistically significant differences were found among the six *B. peruviensis* strains in either leaves or stems (*P* > 0.05; **Figure 1**).

Among the tested strains, *B. peruviensis* RGM 570 exhibited the highest colonization rate in leaves and stems (36% in average between both tissues), In contrast RGM 644 showed the lowest endophytic colonization, with an average of 18% in leaves and stems. However, this difference was not statistically significant when compared to the other *Beauveria* strains studied. No endophytic colonization was detected in the roots of blueberry plants inoculated with any of the strains, as no fungal growth was observed or re-isolated from root tissues.

### 3.2 Persistence of endophytic strains of *B. peruviensis* in blueberry plants

The endophytic colonization rates for each of the six *B. peruviensis* strains in leaves, four weeks after inoculation, ranged from 12% to 48%, with no significant differences among strains (*P* > 0.05). However, strain RGM 644 (12%) exhibited a 36-percentage-point lower colonization rate compared to strain RGM 547, which had the highest colonization rate (48%) after four weeks of fungal inoculation.

After eight weeks, the strains formed two distinct groups: one consisting of strains that maintained endophytic colonization (RGM 547, RGM 557, and RGM 570) with an average colonization rate of 21.3% on leaf-tissues sections, and another comprising strains that showed no colonization (RGM 644, RGM 657, and RGM 731). These groups remained consistent at the 12-week assessment, with an average colonization rate of 16% observed on leaf tissues section of the first group. The RGM 547 strain exhibited a colonization rate of 48% at four weeks, which decreased to 18% at eight weeks, with no significant differences between these two periods. However, both the four and eight-week measurements were significantly different (*P* < 0.05) from the 12-week measurement, where leaf-tissue colonization was reduced at 14%. Similarly, strain RGM 557 exhibited a colonization rate of 38% at four weeks, which decreased to 24% and 22% at eight and twelve weeks, respectively, with no significant differences across time points. A similar trend was observed for strain RGM 570, which reached 46% at four weeks and declined to 22% and 12% at eight and twelve weeks, respectively.

**Figure 2:**
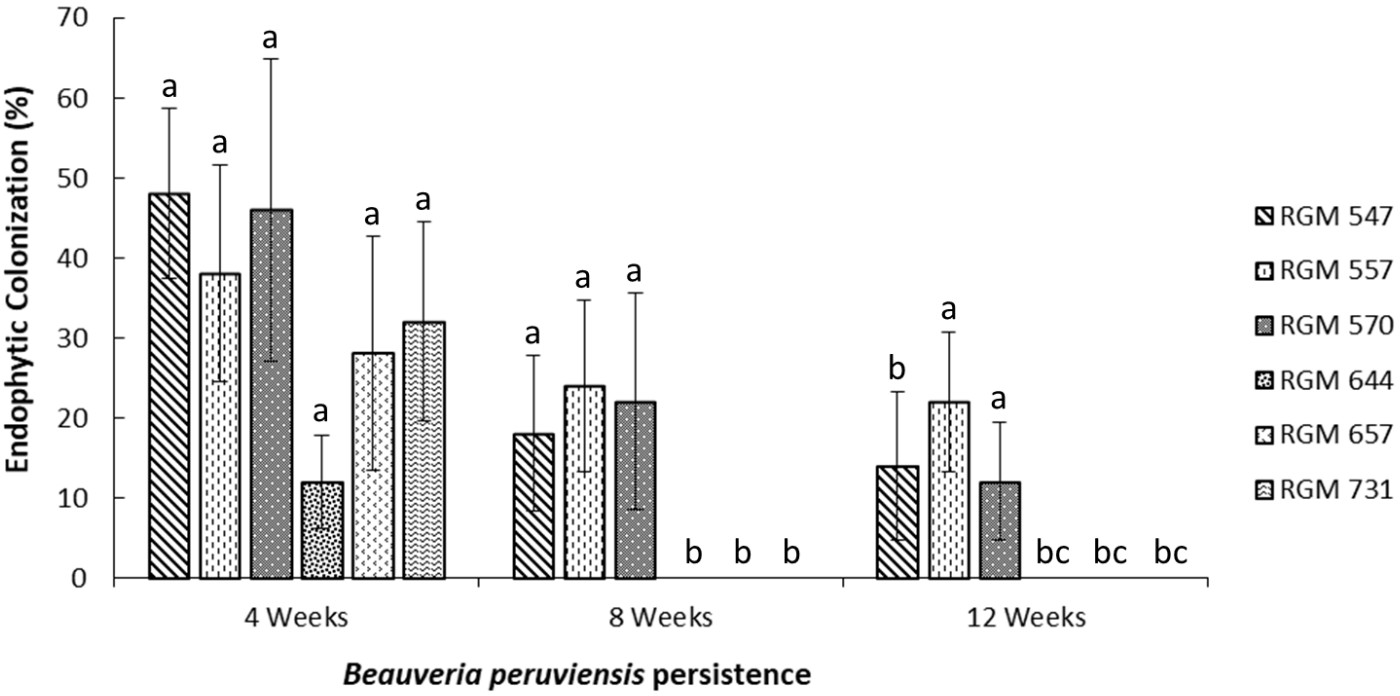
Percentages of endophytic colonization by six *Beauveria peruviensis* strains in the leaves of one-year-old 'Duke' blueberry plants at four, eight, and twelve weeks after foliar inoculation of the strains. Columns represent treatment means, and the bars above them indicate the standard error associated with each mean. Different letters in bars of the same color indicate significant differences according to Friedman's nonparametric repeated-measures test (*P* < 0.05).

Strains RGM 644, RGM 657, and RGM 731 showed an average colonization rate of 24% at four weeks. However, they did not exhibit detectable plant tissue colonization in subsequent evaluations (**Figure 2**).

### 3.3 *In vitro* antagonism of endophytic strains of *B. peruviensis* against *N. mesopotamica*

**Figure 3:**
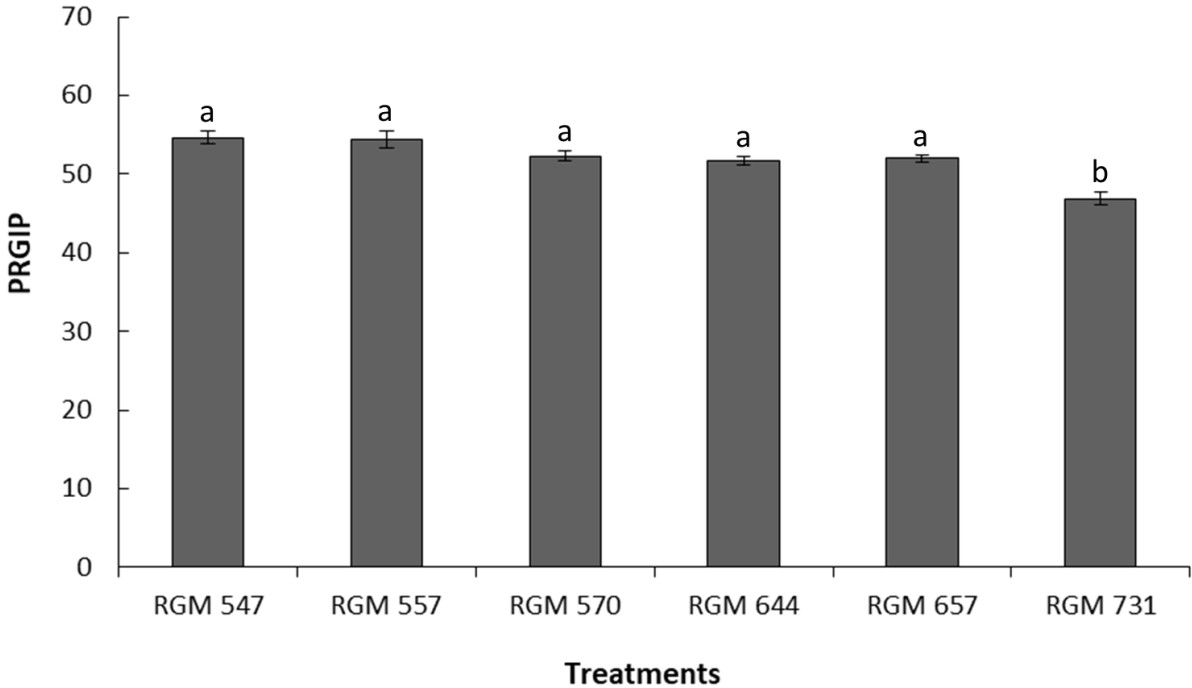
Percentage of radial growth inhibition of pathogen (PRGIP) *Neopestalotiopsis mesopotamica* by *Beauveria peruviensis* strains under *in vitro* co-culture on PDA medium. Plates were incubated in darkness at 25 ± 2°C for seven days. Different letters above the bars indicate significantly significant differences according to Tukey’s *post hoc* test (*P* < 0.05).

The six evaluated strains of *B. peruviensis* exhibited inhibition of pathogen radial growth, ranging from 46.9% to 54.7% (**Figure 3**). RGM 547 was the strain with the highest PRGIP (54.7%), but this was not significantly different from strains RGM 557, RGM 570, RGM 644, and RGM 657, which also did not differ significantly from one another. In contrast, strain RGM 731 exhibited the lowest PRGIP (46.9%), with significant differences (*P* < 0.05) compared to all other evaluated strains (**Figure 3**).

### 3.4 *In vivo* antagonism of endophytic *B. peruviensis* strains against *N. mesopotamica* in blueberry plants

**Figure 4:**
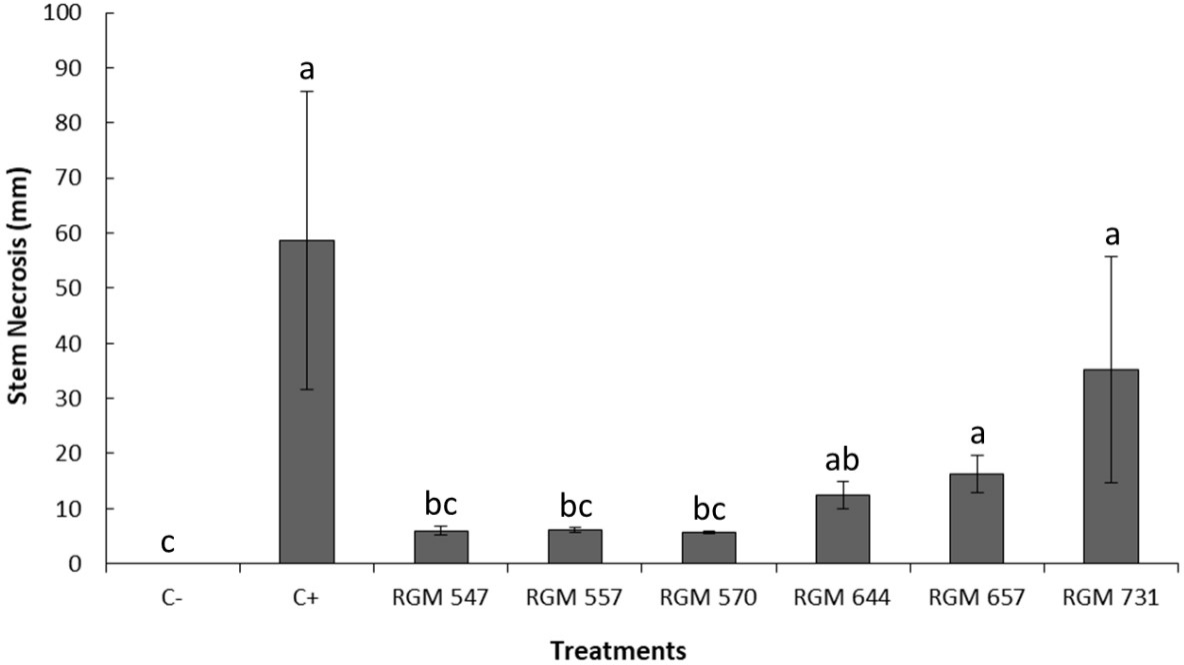
Necrosis length observed in the stems of five-month-old ‘Duke’ blueberry plants four weeks after *Beauveria peruviensis* strain application to the foliage and 23 days after inoculation with *Neopestalotiopsis mesopotamica* strain RGM 3491 (C+). The symbol (C-) represents plants not inoculated with *Neopestalotiopsis* species. Different letters above the columns indicate significant differences according to the nonparametric Kruskal-Wallis test followed by multiple pairwise comparisons (*P* < 0.05). Columns represent treatment means, and the bars above them show the standard error associated with each mean.

The necrosis length observed in the stems of five-month-old ‘Duke’ blueberry plants showed significant differences among *B. peruviensis* strains (*P* < 0.05; **Figure 4**). The positive control (C+), inoculated only with *N. mesopotamica* RGM 3491, exhibited the greatest stem necrosis (58.6 mm), being significantly different from strains RGM 547, RGM 557, and RGM 570 (*P* < 0.05). Plants inoculated with strain RGM 547 had the shortest necrosis length (6 mm), although this was not significantly different from the level of damage observed in plants inoculated with RGM 557, RGM 570, RGM 644, or the non-inoculated control (C-). RGM 547, RGM 557, and RGM 570 showed significant differences compared to strains RGM 657, and RGM 731, which did not differ significantly from the positive control (C+).

**Figure 5:**
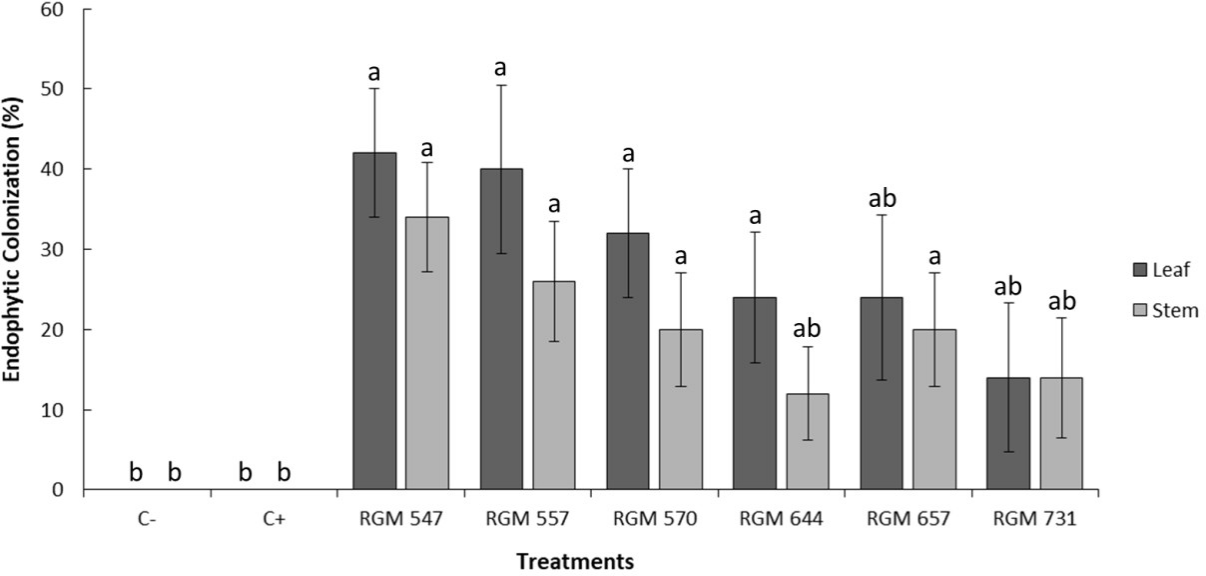
Percentages of endophytic colonization by *Beauveria peruviensis* in leaves and stems of five-month-old ‘Duke’ blueberry plants, four weeks after strain application and 23 days after inoculation with *Neopestalotiopsis mesopotamica* strain RGM 3491 (C+). The symbol (C-) represents plants not inoculated with *N. mesopotamica*. Different letters above the columns indicate significant differences (*P* < 0.05) according to the nonparametric Kruskal-Wallis test followed by multiple pairwise comparisons. Columns represent treatment means, and the bars above them show the standard error associated with each mean.

In parallel with the necrosis length assessment, leaf and stem isolations were performed to confirm the endophytic presence of *Beauveria* in plants leaves and stems treated with different strains (**Figure 5**). The results indicated endophytic colonization in both tissues inoculated with the tested fungal strains, whereas no colonization was detected in C+ or C- (**Figure 5**). Although no major differences were observed in the colonization of leaves and stems, strains RGM 644, RGM 657, and RGM 731, which exhibited lower colonization rate in these tissues, tended to have greater stem necrosis (**Figure 5**).

When stem necrosis data were correlated with the percentage of *Beauveria* isolation in this *in vivo* antagonistic control experiment, a significant inverse relationship was observed between endophytic colonization in leaves (r_s_ = -0.64; *P* < 0.0001) and stems (r_s_ = -0.40; *P* = 0.018).

**Figure 6:**
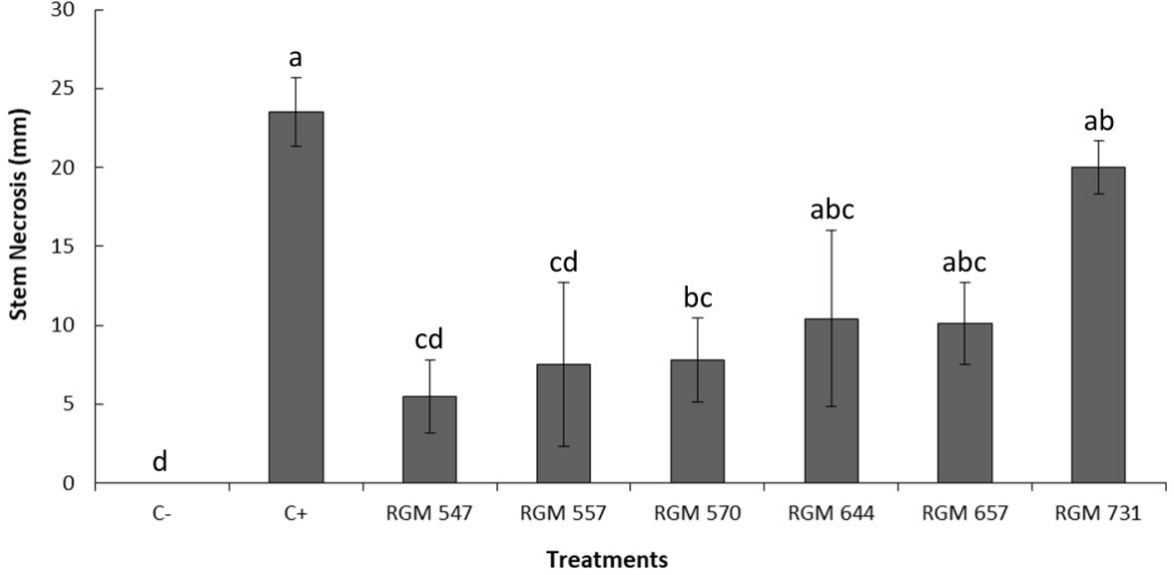
Necrosis length observed in the stems of one-year-old ‘Duke’ blueberry plants four weeks after *Beauveria peruviensis* strain application to the foliage and 23 days after inoculation with *Neopestalotiopsis mesopotamica* strain RGM 3491 (C+). The symbol (C-) represents plants not inoculated with *Neopestalotiopsis* species. Different letters above the columns indicate significant differences according to the nonparametric Kruskal-Wallis test followed by multiple pairwise comparisons (*P* < 0.05). Columns represent treatment means, and the bars above them show the standard error associated with each mean.

**Figure 7:**
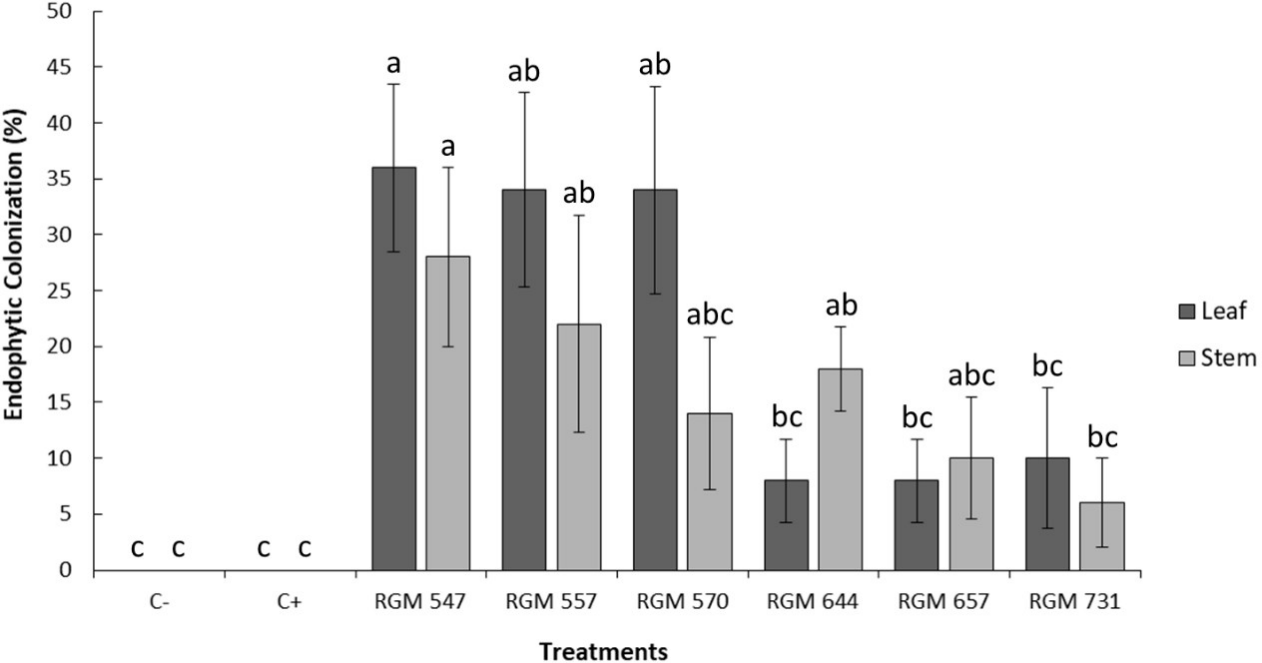
Percentages of endophytic colonization by *Beauveria peruviensis* strain RGM 3491 (C+). The symbol (C-) represents plants not inoculated with *Neopestalotiopsis mesopotamica* strain RGM 3491 (C+). The symbol (C-) represents plants not inoculated with *N. mesopotamica*. Different letters above the columns indicate significant differences (*P* < 0.05) according to the nonparametric Kruskal-Wallis test followed by multiple pairwise comparisons. Columns represent treatment means, and the bars above them show the standard error associated with each mean.

In the evaluation of the *in vivo* antagonism of *B. peruviensis* strains against *N. mesopotamica* in blueberry plants one year old, significant differences were observed in the length of necrosis in the inoculated stems (*P* < 0.05; **Figure 6**). The positive control, inoculated only with *N. mesopotamica* (C+), exhibited the greatest necrosis extension in the stems (23.5 mm), showing significant differences compared to strains RGM 547, RGM 557, RGM 570, and the negative control (C-). In contrast, plants inoculated with *B. peruviensis* strains RGM 547 and RGM 557 exhibited the lowest average necrosis length (5.5 mm and 7.5 mm, respectively), with significant differences compared to C+ and strain RGM 731 (**Figure 6**). Strain RGM 570 also significantly reduced stem necrosis (7.5 mm and 7.8 mm, respectively), differing from C +. On the other hand, strains RGM 644, RGM 657, and RGM 731 did not differ from C+ and were significantly different from C-. In parallel, leaf and stem isolations were performed to confirm the endophytic colonization of *Beauveria* in one-year-old plants inoculated with the evaluated strains (**Figure 7**). All studied strains successfully colonized both tissues endophytically, whereas no colonization was detected in the controls inoculated with *N. mesopotamica* (C+) or in the non-inoculated controls (C-). Endophytic colonization of *B. peruviensis* ranged from 8% to 36% in leaves and from 6% to 28% in stems. As observed in five-month-old blueberry plants, two significantly different groups were formed based on leaf colonization. Strains RGM 547, RGM 557, and RGM 570 exhibited colonization levels between 34% and 36% in leaves, whereas strains RGM 644, RGM 657, and RGM 731 showed colonization levels between 8% and 10%, with no significant differences from the two untreated controls. In stems, colonization by *B. peruviensis* strains did not exhibit significant differences, except for RGM 731, which differed from RGM 547, the strain with the highest colonization rate (28%) among those evaluated. Only RGM 547, RGM 557, and RGM 644 showed endophytic colonization levels significantly different from those recorded in both controls (C+ and C-) (**Figure 7**). When correlating necrosis length with the percentage of *Beauveria* isolation in this *in vivo* antagonism experiment, a significant inverse relationship was observed for endophytic colonization in leaves (r_s_ = -0.98; *P* = 0.0001), while the correlation in stems was not statistically significant (r_s_ = -0.75; *P* = 0.06).

## 4 Discussion

### 4.1 Endophytic colonization of *Beauveria peruviensis* strains in blueberry plants

Native strains of *B. peruviensis*, obtained from soils in various ecosystems in Chile demonstrated the ability to endophytically colonize five-month-old blueberry (*V. corymbosum* cv. Duke) plants following artificial inoculation with a foliar-applied conidial suspension. Endophytic colonization rates ranged from 16% to 64% in leaves and from 20% to 32% in stems. These results are comparable to those reported by Barra-Bucarei et al. (2019), in which the same native strains of *B. peruviensis* successfully colonized tomato and chili pepper plants. In herbaceous species such as tomato, leaf and stem colonization rates were similar (Barra-Bucarei et al., 2019). However, in blueberry plants, the highest colonization occurred in leaves, with comparatively lower rates in stems. This discrepancy may be attributed to the progressive lignification of blueberry stem tissues, a characteristic of perennial shrubs, which could hinder or delay the establishment of external endophytes like *B. peruviensis*.

In the *in vivo* antagonism test against *N. mesopotamica* conducted on one-year-old blueberry plants, average colonization levels in stems were even lower, ranging from 6% to 28% (16.3% in average). Similarly, studies on Solanaceae such as tomato has also reported higher colonization rates in leaves than in stems (42% versus 11.7% in average, respectively [Barra-Bucarei et al., 2019]). Additionally, *B. peruviensis* was not isolated from the roots of blueberry plants, in contrast to its behavior in Solanaceous species. This may be explained by the tendency of *Beauveria* colonization to remain localized near the site of inoculation, with limited translocation to distal tissues (Sinno et al., 2021). Furthermore, previous studies have shown that substrate or soil inoculation favors root colonization (Barra-Bucarei et al., 2019; Posada and Vega, 2005). However, this method was not applied in the present study as *N. mesopotamica* primarily affects the aerial parts of blueberry plants.

Previous research suggests that inoculated *Beauveria* species have the potential to migrate through interconnected vascular tissues and systemically colonize the entire plant (Barra-Bucarei et al., 2019; Bamisile et al., 2019; Mantzoukas et al., 2015; Wagner and Lewis, 2000). However, the results presented here contrast with those of Bamisile et al. (2019), who found that while one of the two evaluated *B. bassiana* strains was capable of systemically colonizing leaves, stems, and roots in lemon, the other strain only colonized leaves and stems following foliar application. In blueberries, longer evaluation periods may be necessary to determine whether *B. peruviensis* strains can achieve systemic colonization. Moreover, Resquín-Romero et al. (2016) suggest that full systemic colonization through foliar applications may not be feasible, indicating that colonization may be transient and non-systemic, with limited basipetal movement in blueberry plants. Compared to annual herbaceous plants, reports on the endophytic behavior of entomopathogenic fungi such as *Beauveria* spp. in woody perennials plants are relatively scarce. However, research has documented the ability of *Beauveria* species to endophytically colonize a variety of perennial hosts, including cocoa (Posada and Vega, 2005), date palm (Gómez-Vidal et al., 2006), coffee (Posada et al., 2007), pine (Reay et al., 2010; Brownbridge et al., 2012), grapevine (Jaber, 2015; Rondot and Reineke, 2018), horse chestnut (Barta, 2018), lemon (Bamisile et al., 2019, 2020), and pecan (Ramakuwela et al., 2020). These findings indicate that, although less frequently reported, woody perennials can indeed host *Beauveria* spp. as endophytes, opening new opportunities for their application as biocontrol agents in permanent cropping systems such as blueberry cultivation.

### 4.2 Persistence of endophytic strains of *B. peruviensis* in blueberry plants

This study demonstrated significant variation in the ability of different *B. peruviensis* strains to colonize and persist within blueberry plants after 12 weeks (three months). Although all six strains exhibited some level of endophytic colonization at four weeks post-inoculation, only strains RGM 547, RGM 557, and RGM 570 maintained consistent colonization over time. Among them, RGM 557 and RGM 570 showed the highest and most sustained colonization rates, suggesting that these strains possess traits that enhance their long-term persistence in blueberry tissues. These findings highlight the importance of selecting *Beauveria* strains not only for their initial colonization efficiency but also for their capacity to persist endophytically, which is critical for successful applications in plant-microbe interactions and biological control strategies.

*Beauveria peruviensis* strains showed higher colonization levels one month after application to blueberry plants. However, in some strains, colonization declined or became undetectable at eight and twelve weeks post-inoculation. This pattern is consistent with findings by Bamisile et al. (2020), who reported that the highest rates of endophytic recovery and fungal migration were observed in younger lemon seedlings, with both establishment and fungal effects decreasing as the plants age. Our results support these observations, suggesting that physiological changes associated with plant as maturation may influence the persistence of endophytes. Nonetheless, long-term colonization is possible in woody species, as demonstrated by Brownbridge et al. (2012), who reported endophytic presence of *B. bassiana* in pine over a period of nine months. How multiple applications of *Beauveria* spp. following the initial treatment could affect endophytic colonization over an extended period in blueberry need to be considered in future research. Especially considering that, in practical field conditions, it is important to determine whether reapplication at intervals (e.g., 15 days or one month after the first treatment) is necessary to enhance and sustain the fungicidal effect of *B. peruviensis*.

### 4.3 Inhibition of the pathogen *N. mesopotamica* by endophytic strains of *B. peruviensis.*

*Beauveria peruviensis* strains exhibited high levels of pathogen inhibition, with an average of 52% (range = 46.9%–54.7%), compared to their inhibitory effect on *Botrytis cinerea*, which ranged from 30% to 39% (Barra-Bucarei et al., 2019). This inhibitory capacity may be attributed to the ability of these fungi to produce a broad spectrum of bioactive metabolites, including bassianolide, bassianin, beauveriolide, bassiacridine, cyclosporine, oosporein, and beauvericin, the latter two having demonstrated antifungal activity (Russo et al., 2024). The diffusion of these compounds into the culture medium influences the growth of pathogenic fungi (Barra-Bucarei et al., 2019; Russo et al., 2023). However, the presence of these compounds in the strains used in this study, as well as in those previously assessed in Solanaceae by Barra-Bucarei et al. (2019), has not yet been confirmed and requires further investigation.

Species of *Beauveria* have demonstrated *in vitro* antagonism against a variety of pathogens (Russo et al., 2024; Deb et al., 2023; Barra-Bucarei et al., 2019; Deb and Dutta, 2021; Ownley et al., 2008; Jaber, 2015; Ownley et al., 2004; Jaber and Ownley, 2018). In addition to the *in vitro* antibiosis observed in the present study, these endophytes may also function as opportunistic competitors, inhibiting pathogen growth by occupying space and consuming nutrients, thereby restricting the development and proliferation of fungal pathogens (Barra-Bucarei et al., 2019). Therefore, evaluating the antagonistic potential of *Beauveria* strains in plants was deemed both necessary and highly valuable for understanding their biocontrol capabilities.

Endophytic strains of *B. peruviensis* applied to foliage effectively reduced the damage caused by *N. mesopotamica* in blueberry stems inoculated through wounds, in both five-month-old and one-year-old plants of the Duke cultivar. However, the reduction in damage was more pronounced in five-month-old plants one month after inoculation with *N. mesopotamica* compared to one-year-old plants. Similarly, *N. mesopotamica* exhibited greater aggressiveness in five-month-old plants, as evidenced by the control group, which developed stem necrosis with an average length of 58.6 mm, whereas in one-year-old plants of the same cultivar, necrosis length averaged only 23.5 mm. Bamisile et al. (2020) reported that the highest rates of endophytic recovery and fungal migration were observed in younger lemon seedlings, with both establishment and fungal effects decreasing as the plants age. Our results support these observations, suggesting that physiological changes associated with plant maturation may influence the persistence of endophytes. Probably, early colonization of the plant by *B. peruviensis* has less defense response activity in the plant facilitating the colonization or cuticle could be less stronger to reduce fungal colonization in younger plants.

*Neopestalotiopsis mesopotamica* is a phytopathogenic fungus known to cause severe diseases in agricultural crops, including strawberry (Hidrobo-Chávez et al., 2022; Intriago-Reyna et al., 2021), tomato (Ayoubi and Soleimani Pari, 2016), and blueberry (Barrera-Merino et al., 2025). The genus *Neopestalotiopsis* is geographically widespread and capable of infecting a broad range of host’s species (Liu et al., 2022; Maharachchikumbura et al., 2014). In blueberry, species of *Neopestalotiopsis* are among the primary causal agents of stem blight, stem cankers, and dieback (Barrera-Merino et al., 2025; Dietsch et al., 2025; Borrero et al., 2018; Lee et al., 2019; Rodríguez-Gálvez et al., 2020; Jevremović et al., 2022; Santos et al., 2022; Shi et al., 2017; González et al., 2012; Espinoza et al., 2008; Chen et al., 2016), all of which can significantly reduce plant productivity and fruit quality. For example, diagnostic records from the Plant Pathology Laboratory at the Chilean Institute of Agricultural Research (INIA-Quilamapu) indicate that, between 2019 and 2024, 39.2% of 178 blueberry plant samples showing symptoms of stem blight, stem cankers, or dieback were diagnosed as infected by *Neopestalotiopsis* species.

The severity of necrosis, expressed as the stem length affected by the pathogen in blueberry plants treated with *B. peruviensis*, suggests significant antifungal activity of these strains. RGM 547, RGM 557, and RGM 570 exhibited a remarkable reduction in necrosis length compared to the positive control inoculated with *N. mesopotamica* alone. This finding aligns with previous studies that highlight the potential of endophytes as biocontrol agents, demonstrating their ability to mitigate the impact of phytopathogenic fungi in various crops (Iida et al., 2023; Barra-Bucarei et al., 2019; Lu et al., 2021; Ahmed et al., 2020). In woody crops, fungi such as *Trichoderma* specie has been effective in controlling *Cryphonectria parasitica* in chestnut (Murolo et al., 2019) and *Eutypa lata* and *Neofusicoccum parvum* in vineyards, both pathogens associated with grapevine trunk diseases (Blundell and Eskalen, 2022; Travadon et al., 2023). Additionally, *Aureobasidium pullulans*, a yeast, has been shown to control *N. parvum* in apple trees (Rusin et al., 2019), while *Clonostachys rosea* inhibits *E. lata* and *Calosphaeria pulchella* in cherry trees, and *E. lata* and *N. parvum* in almond trees (Travadon et al., 2023). These studies underscore the crucial role of fungal endophytes in protecting woody plants, which are frequently exposed to phytopathogenic fungi that threaten their productivity and longevity. The ability of *B. peruviensis* strains to endophytically colonize blueberry plants and confer protection, as evidenced by reduced necrosis, suggests that this fungus could serve as a promising alternative for managing pathogens affecting woody species.

The mechanisms underlying this biocontrol capacity remain to be elucidated. However, it has been proposed that species of the *Beauveria* genus act by competing for resources and space within the host (Barra-Bucarei et al., 2019; Russo et al., 2023). In this context, *B. peruviensis* hyphae may occupy plant tissues, thereby restricting the ability of the pathogen’s ability to establish and proliferate (Ownley et al., 2008). Additionally, this behavior could suggest potential mycoparasitism, a mechanism previously described for *B. bassiana*, where the endophytes structures directly interfere with those of the pathogen, weakening it and reducing its ability to cause damage (Russo et al., 2023). However, further studies are needed to confirm this mechanism in *B. peruviensis* against *N. mesopotamica*. Another potential biocontrol mechanism could involve the induction of defense responses in blueberry plants by *B. peruviensis*. This endophyte may trigger induced systemic resistance (ISR) in plants, leading to the activation of defense mechanisms that suppress *N. mesopotamica* while allowing the beneficial microorganism to persist. The ability of microorganisms to induce ISR in blueberries has been previously demonstrated for *Schizophyllum commune* G18, which was found to reduce root rot caused by *Fusarium commune* in blueberries. This was achieved by producing hydrolytic enzymes that degrade pathogenic fungal hyphae and by enhancing the activity of antioxidant enzymes, such as superoxide dismutase (SOD), peroxidase (POD), and catalase (CAT) in the leaves and roots of inoculated blueberry seedlings (Li et al., 2024). These enzymes serve as indicators of plant resistance responses to pathogens (Li et al., 2024). Overall, multiple mechanisms may contribute to the protective effects of endophytes in blueberries, including the production of antimicrobial metabolites, mycoparasitism, competition for resources and space, and the activation of systemic defense responses by *B. peruviensis* strains. These findings are consistent with previous studies on other endophytes within this genus (Jaber and Ownley, 2018; Barra-Bucarei et al., 2019; Qin et al., 2021; Ahmed et al., 2020). However, these mechanisms need to be further studied in the *B. peruviensis* - N*. mesopotamica* pathosystem.

The observed correlation between greater endophytic colonization and reduced stem necrosis supports the hypothesis that the presence of the endophyte not only exerts direct antagonistic effects but may also trigger broader defense responses in the host. This aligns with reports suggesting that endophytes can induce the production of secondary metabolites or activate signaling pathways that enhance plant resistance to pathogen attacks (Lu et al., 2021; Qin et al., 2021). Future research should aim to elucidate the specific mechanisms involved, evaluate the impact of different inoculation methods, assess the efficacy against other blueberry pathogens, and examine interactions under diverse environmental conditions to optimize their biocontrol potential.

## 5 Conclusions

Chilean native strains of *Beauveria peruviensis* exhibit significant potential as biocontrol agents against *Neopestalotiopsis mesopotamica* both *in vitro* and in blueberry plants. These *B. peruviensis* strains demonstrated the ability to endophytically colonize blueberry leaves and stems, with varying levels of persistence over time following foliar application. Strains RGM 547, RGM 570, and RGM 557 displayed the highest colonization capacity and persistence, as well as the greatest reduction in stem necrosis caused by *N. mesopotamica*. The increased endophytic colonization of *B. peruviensis* correlated with a reduction in pathogen-induced stem necrosis. These findings suggest that *B. peruviensis* could serve as a sustainable alternative to synthetic fungicides for managing stem blight disease in blueberries caused by *N. mesopotamica*.

## Data Availability

The raw data supporting the conclusions of this article will be made available by the authors, without undue reservation.
