# Supplementary material for: Endophytic colonization of Beauveria peruviensis and its antagonistic activity against Neopestalotiopsis mesopotamica in blueberry
Source: Front Plant Sci. 2026 May 19;17:1829417. doi: 10.3389/fpls.2026.1829417 (PMC13226539; doi:10.3389/fpls.2026.1829417)
Supplement: Supplementary file 1 [file Table1.docx]

Supplementary Material

# Supplementary Figures


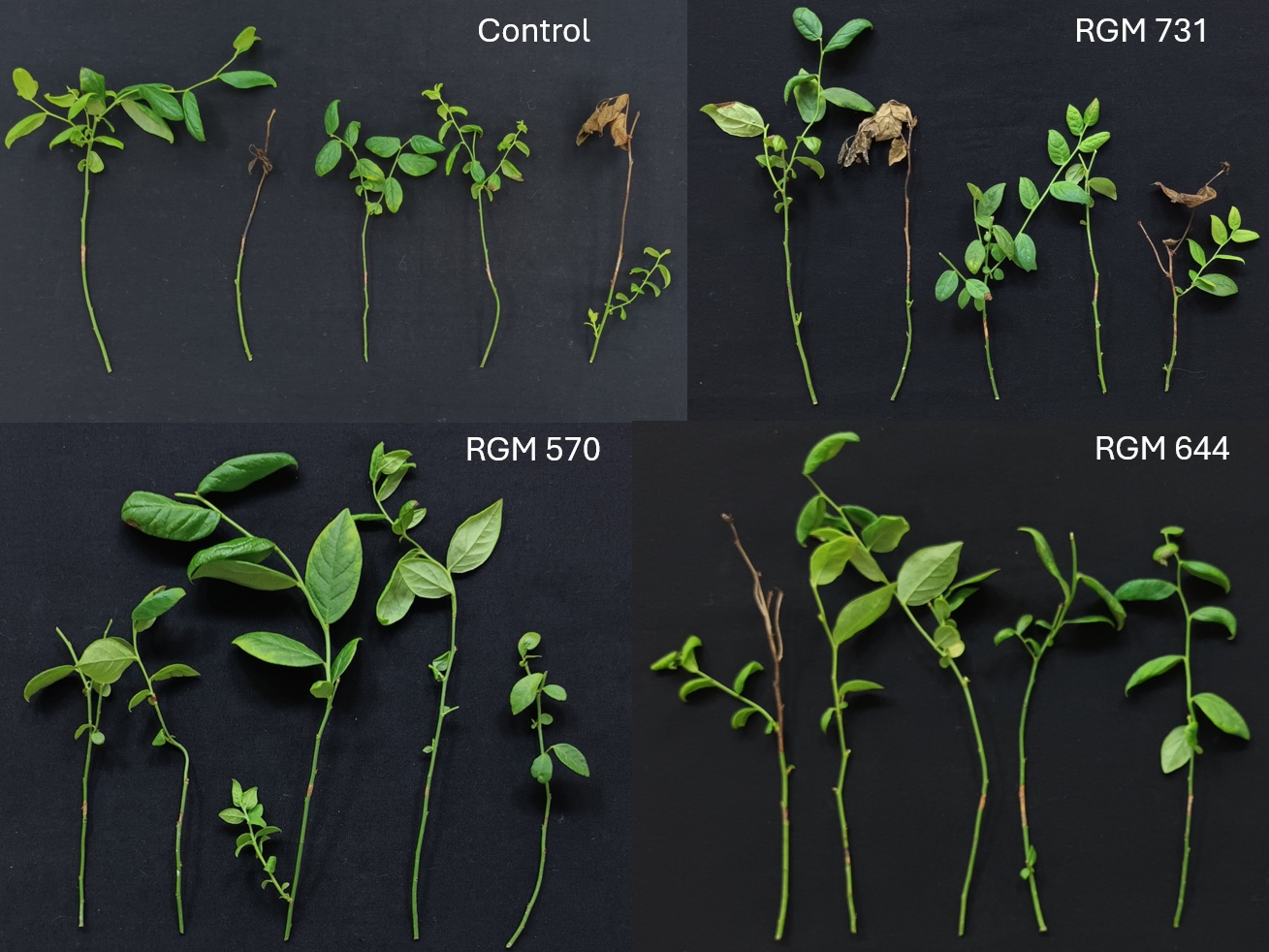


Figure S1. View of stems of five-month-old ‘Duke’ blueberry plants four weeks after foliar application of *Beauveria peruviensis* strain and 23 days after inoculation with *Neopestalotiopsis mesopotamica* strain RGM 3491. Control shows stems of plants untreated with some strains of the fungal antagonist, while RGM 570 shows marked control on plants inoculated with *N. mesopotamica*. RGM 731 did not show an adequate control on the pathogenic fungus, whereas RGM 644 shows light control, which was not statically different from the untreated Control.


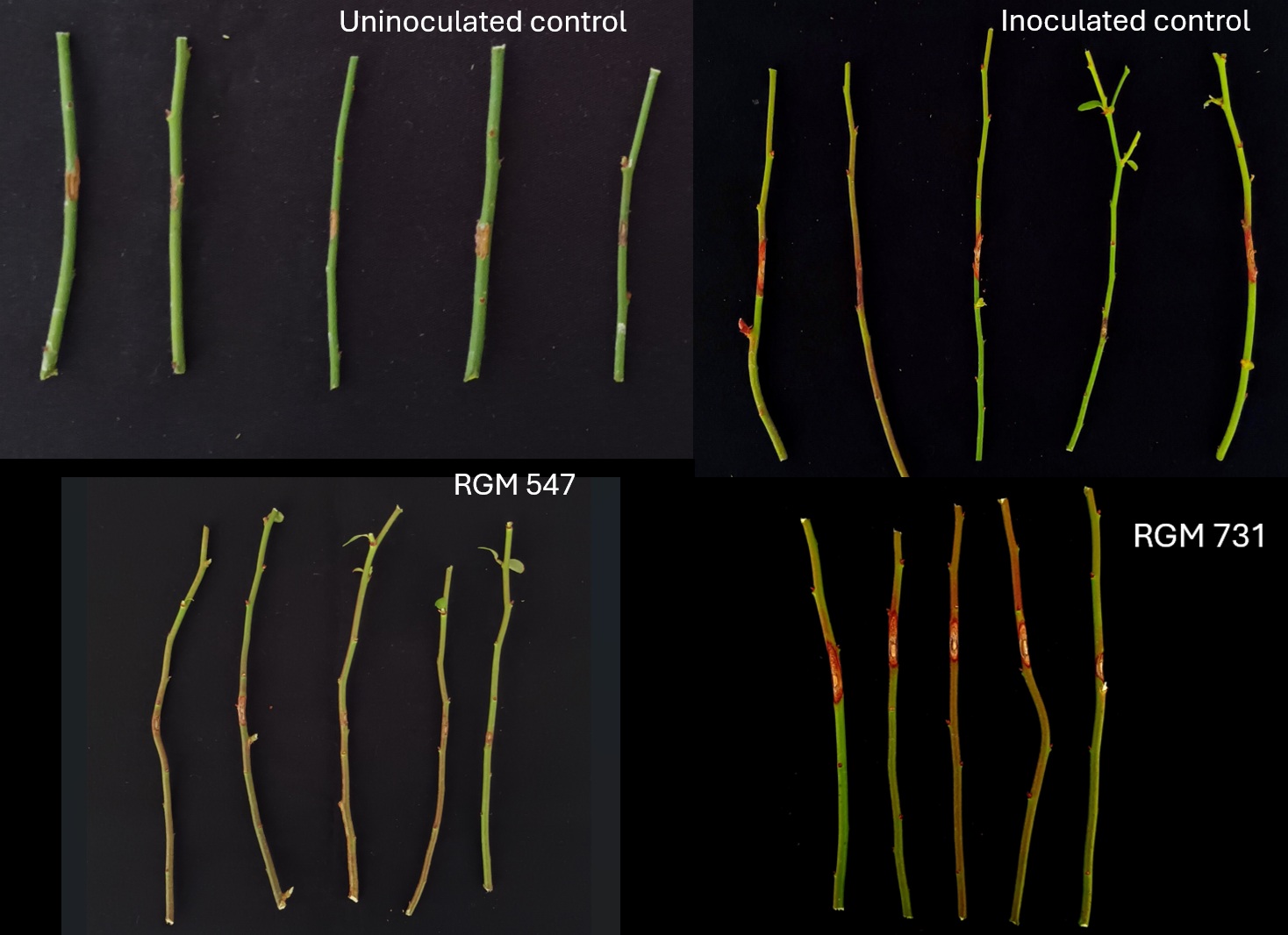


Figure S2. View of stems of one-year-old ‘Duke’ blueberry plants four weeks after foliar application of *Beauveria peruviensis* strain and 23 days after inoculation with *Neopestalotiopsis mesopotamica* strain RGM 3491. Uninoculated control shows stems of plants only inoculated with sterile distillated water, whereas the inoculated control was only with *N. mesopotamica*. RGM 547 shows marked control on plants inoculated, while RGM 731 did not show adequate control on the pathogenic fungus, which was not statically different from the untreated Control.

**
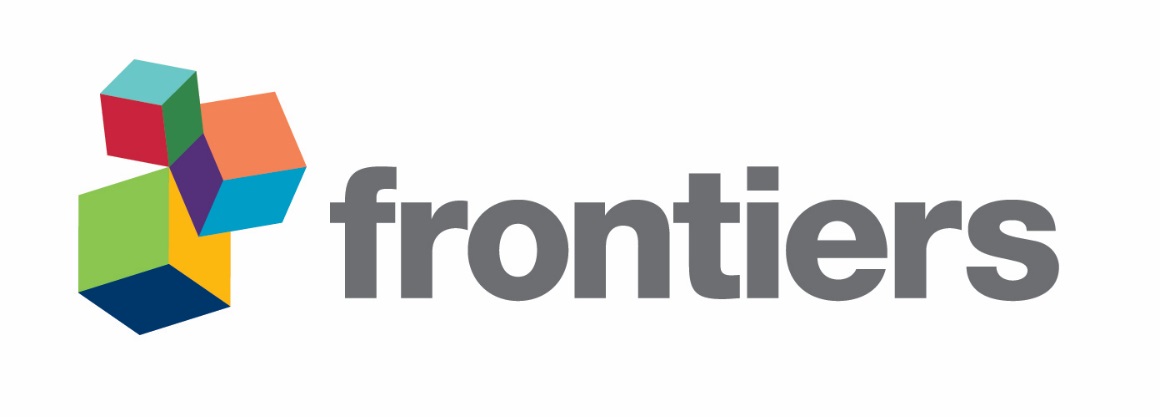
**
